# Artificial intelligence for optimum tissue excision with indocyanine green fluorescence angiography for flap reconstructions: Proof of concept

**DOI:** 10.1016/j.jpra.2024.07.014

**Published:** 2024-07-31

**Authors:** Ashokkumar Singaravelu, Jeffrey Dalli, Shirley Potter, Ronan A. Cahill

**Affiliations:** a UCD Centre for Precision Surgery, University College Dublin, Ireland; b Department of Plastic and Reconstructive Surgery, Mater Misericordiae University Hospital, Dublin 7, Ireland; c Department of Surgery, Mater Misericordiae University Hospital, Dublin 7, Ireland

**Keywords:** Indocyanine green, Fluorescence imaging, Flap perfusion, Artificial intelligence, Expert interpretation

## Abstract

**Background:**

Indocyanine green fluorescence angiography (ICGFA) is gaining popularity as an intraoperative tool to assess flap perfusion. However, it needs interpretation and there is concern regarding a potential for over-debridement with its use. Here we describe an artificial intelligence (AI) method that indicates the extent of flap trimming required.

**Methods:**

Operative ICGFA recordings from ten consenting patients undergoing flap reconstruction without subsequent partial/total necrosis as part of an approved prospective study (NCT 04220242, Institutional Review Board Ref:1/378/2092), provided the training-testing datasets. Drawing from prior similar experience with ICGFA intestinal perfusion signal analysis, five fluorescence intensity and time-related features were analysed (MATLAB R2024a) from stabilised ICGFA imagery. Machine learning model training (with ten-fold cross-validation application) was grounded on the actual trimming by a consultant plastic surgeon (S.P.) experienced in ICGFA. MATLAB classification learner app was used to identify the most important feature and generate partial dependence plots for interpretability during training. Testing involved post-hoc application to unseen videos blinded to surgeon ICGFA interpretation.

**Results:**

Training:testing datasets comprised 7:3 ICGFA videos with 28 and 3 sampled lines respectively. Validation and testing accuracy were 99.9 % and 99.3 % respectively. Maximum fluorescence intensity identified as the most important predictive curve feature. Partial dependence plotting revealed a threshold of 22.1 grayscale units and regions with maximum intensity less then threshold being more likely to be predicted as “excise”.

**Conclusion:**

The AI method proved discriminative regarding indicating whether to retain or excise peripheral flap portions. Additional prospective patients and expert references are needed to validate generalisability.

## Introduction

Indocyanine green fluorescence angiography (ICGFA) is gaining popularity as a tool to assess flap perfusion, in particular to help identify those likely to undergo edge necrosis. A recent meta-analysis shows that ICGFA can reduce perfusion-related complication rate.^1^ However, interpretation is subjective and needs experience. Furthermore, there is some concern that ICGFA may guide surgeons to over-debride tissue beyond that deemed necessary by surgeons’ view (which may induce other complications including need for further reconstructions to fit the resulting defect).^2^

This proof-of-concept work developed an artificial intelligence (AI) model that represents how an expert surgeon would interpret ICGFA signals regarding whether to trim or retain the flap edge and, if trimming is necessary, indicate the appropriate extent.

## Methods

This work was performed as part of a registered study (NCT 04220242, Institutional Review Board approval reference 1/378/2092).

### Patients

ICGFA recordings from consenting patients undergoing flap procedures in a single university hospital without subsequent postoperative partial/total flap necrosis were used for training-testing. ICG dye (0.1 mg/kg in 1 patient, 0.25 mg/kg in the rest) was administered intravenously and fluorescence intensity visualised using a commercial imager (EleVision™ IR Platform, Medtronic, Dublin, Ireland). Intraoperative decisions regarding flap trimming (including extent) were made after subjective ICGFA assessment by an experienced surgeon (S.P.) and recorded prospectively.

### Algorithm development

A modelling approach previously utilised successfully with intestinal perfusion analysis for transection site selection during colorectal surgery was utilised.^3^ ICGFA imagery were stabilised using affine geometric transformation and divided into 72×96 grids (MATLAB R2024a). Five fluorescence intensity curve features (maximum fluorescence intensity, upslope, time to reach maximum fluorescence intensity and 50 % of maximum intensity, and time ratio) were extracted from each grid. For data augmentation, four lines of different lengths and positions across the flap length were sampled in each video. This involved creating 70-pixel high rectangular regions centered on each line, calculating median values at every coordinate, and condensing the data into a single line representation. The flap region resected by the operating surgeon (S.P.) was labelled “excise” and the region of the flap retained labelled “retain”. The data was imported into MATLAB Classification Learner App and models trained with 10-fold cross validation. The best performing model by accuracy progressed to unseen testing with predictions overlaid on white light imagery, using green to indicate “retain” and red to indicate “excise”. AI predictions were compared to actual in-theatre ICGFA interpretations including any flap trimming with true positive rates calculated for each label.

### Feature importance and interpretability

Feature importance scores were generated for curve features using the reliefF algorithm allowing partial dependence plot generation for the highest importance scoring feature for model interpretation.

## Results

Training involved seven patient videos (28 lines of flap length, 1413 grids) and testing involved three patient videos (three lines, 149 grids)(Table S1). Flap edge trimming occurred in four patients (two in both the training and testing sets).

**Figure 1:**
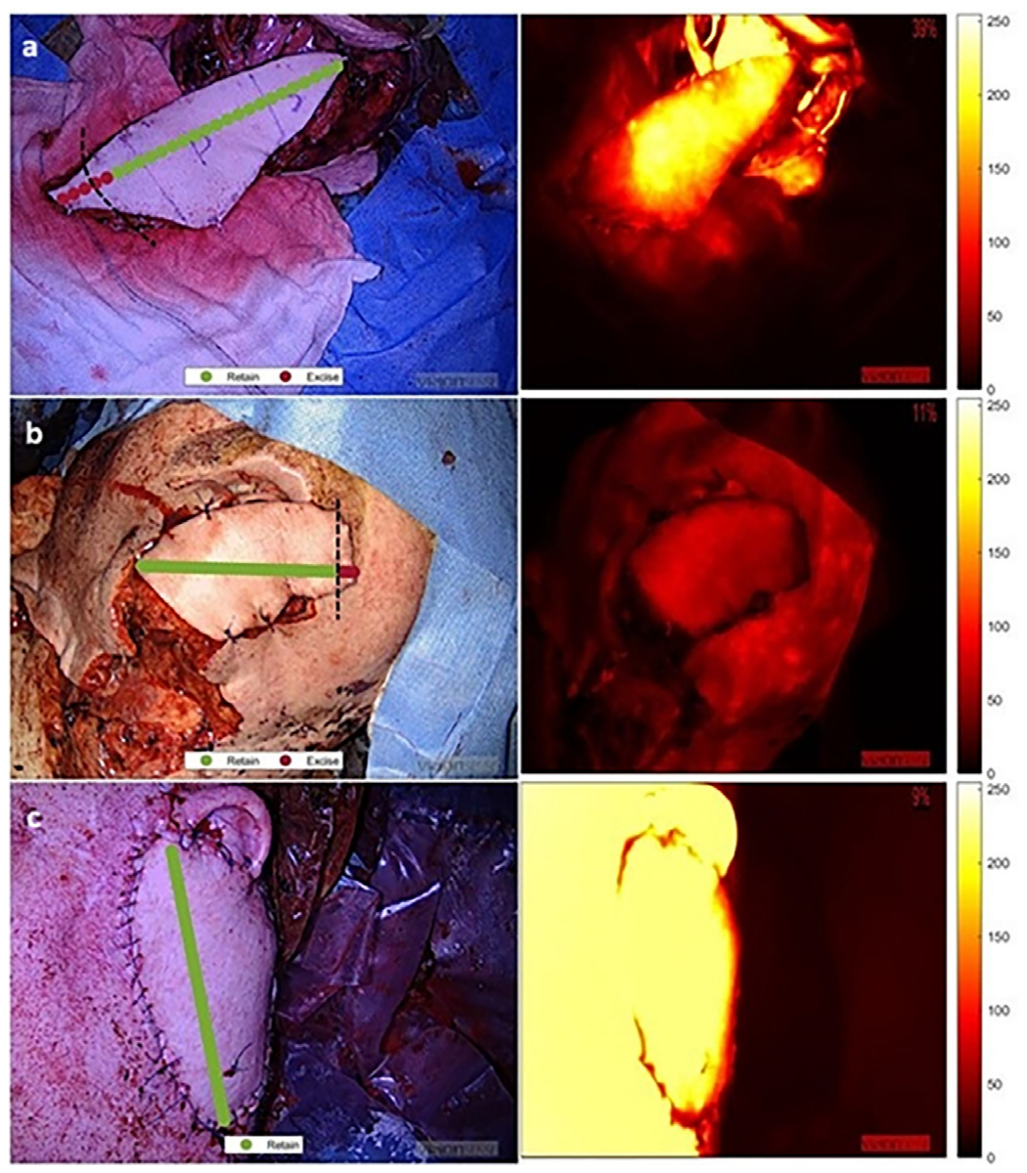
Operative imagery with prediction overlay (left) on three test cases (a-c) alongside maximum fluorescence intensity per pixel heatmap (right). The dotted lines represent the actual location of flap trimming.

Ensemble subspace KNN model had the highest overall validation (99.9 %) and testing accuracy (99.3 %) with the model taking 23.2 s to train. In the validation dataset, the true positive rate for “retain” and “excise” class were 99.9 % (1397/1398) and 100 % (15/15) respectively. In testing, the true positive rate for “retain” and “excise” class were 100 % (138/138) and 90.9 % (10/11) respectively. Figure 1 shows prediction overlays along with maximum intensity per pixel heatmaps.

### Feature importance and interpretability

**Figure 2:**
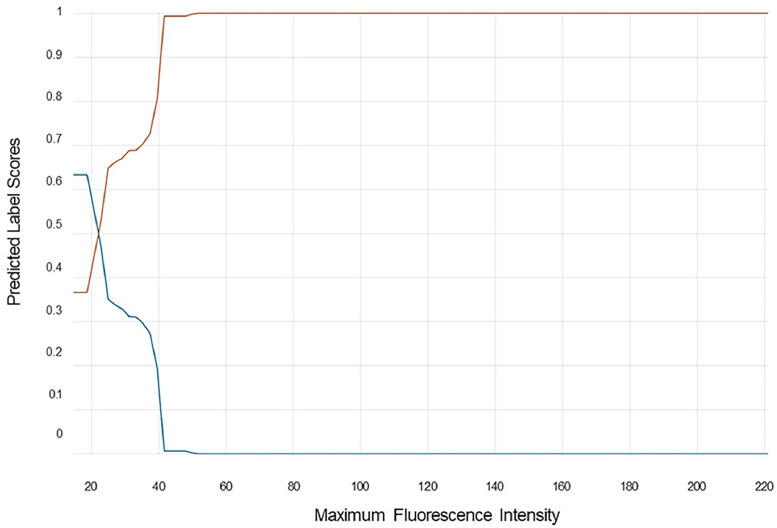
Partial dependence plot of the highest importance scoring feature (maximum fluorescence intensity). Blue = Excise, Orange = Retain.

Maximum fluorescence intensity was identified as the most important feature for predictions by reliefF algorithm (supplementary Figure S1). The partial dependence plot (Figure 2) shows that regions with maximum fluorescence intensity less than 22.1 grayscale units were more likely to be predicted as “excise”.

## Discussion

ICGFA allows for real-time visualisation of flap perfusion including in its distal portions. Alongside expert user experience, its useability and reproducibility by other surgeons needs focus. This work provides a method to recreate expert decision regarding flap edge trimming which could be beneficial for novice or occasional users. We also show that maximum intensity per pixel heatmap alone may be used to predict location of trimming. This aligns with other reports of how surgeons interpret ICGFA subjectively (e.g. Namin et al. resected flap where the skin no longer appeared green/fluorescent).^4^ There is variability in the literature regarding the threshold guiding excision, with some studies using relative thresholds (≤ 25–30 %) and others using absolute intensity (6 grayscale units).^1^^,^^5^ The work is limited by being relatively small and retrospective. However, similar methodology has already been validated regarding bowel perfusion including prospectively.^3^ The model was trained on only one ICGFA expert and would seem likely to perform better, including in generalisablity to other surgeons, with more patient videos and ICGFA expert interpretations.

## Conclusion

This work indicates successful AI-ICGFA prediction of flap trimming where necessary with maximum fluorescence intensity as the most important predictive factor. Next step development will focus on additional training data and prospective in-theatre trialling.

## Funding

This study was supported by the Disruptive Technologies Innovation Fund as awarded by The Government of Ireland via Enterprise Ireland.

## Ethical approval

Computational explorations and software development were performed as part of a registered study (NCT 04220242, Institutional Review Board approval reference 1/378/2092).

## Declaration of competing interest

Professor Ronan A Cahill is named on a patent filed in relation to processes for visual determination of tissue biology, receives speaker fees from Stryker Corp and Ethicon/J&J, consultancy fees from Arthrex, Astellas, Diagnostic Green and Touch Surgery (Medtronic), research funding from Intuitive Corp and Medtronic as well as, previously, from the Irish Government (DTIF) in collaboration with IBM Research in Ireland and Deciphex, and from EU Horizon 2020 in collaboration with Palliare and, currently, from Horizon Europe in collaboration with Arctur. Dr J Dalli was employed as a researcher in the DTIF and is a recipient of the TESS scholarship. Other authors have no industry disclosures to report.

## Appendix Supplementary materials

Image, application 1
