# Supplementary material for: Artificial intelligence for optimum tissue excision with indocyanine green fluorescence angiography for flap reconstructions: Proof of concept
Source: JPRAS Open. 2024 Jul 31;41:389–93. doi: 10.1016/j.jpra.2024.07.014 (PMC11381603; doi:10.1016/j.jpra.2024.07.014)
Supplement: Supplementary file 1 [file mmc1.docx]

**Artificial intelligence for optimum tissue excision with indocyanine green fluorescence angiography for flap reconstructions: proof of concept**

Ashokkumar Singaravelu^1^, Jeffrey Dalli^1,3^, Shirley Potter^2^, Ronan A. Cahill^1,3^

^1^UCD Centre for Precision Surgery, University College Dublin, Ireland.

^2^Department of Plastic and Reconstructive Surgery, Mater Misericordiae University Hospital, Dublin 7, Ireland.

^3^Department of Surgery, Mater Misericordiae University Hospital, Dublin 7, Ireland.

**Corresponding author.**

Prof Ronan A. Cahill, 47 Eccles Street, Dublin 7, Ireland.

Email: [ronan.cahill@ucd.ie](mailto:ronan.cahill@ucd.ie) Telephone: 00353 1 716 4597 ORCID ID: 0000-0002-1270-4000

**Supplementary Materials - Index**

| **Supplementary Table** |  |
| --- | --- |
| Table S1 | *pag. 2* |
| Figure S1 | *pag. 3* |

**Table S1.** Patient demographics including reconstructive flap type and postoperative complications.

|  | **Training (n=7)** | **Testing (n=3)** |
| --- | --- | --- |
| **Median (interquartile range) age in years** | 66 (53.5-77) | 66 (61.5-75) |
| **Male:Female** | 3:4 | 3:0 |
| **Flap Reconstructions** | Free deep inferior epigastric perforator flaps (n=3); Occipital scalp flaps (n=2, one pedicle); Free anterolateral thigh flap and Pedicled latissimus dorsi flap (n=1 each) | Free anterolateral thigh flaps (n=2); Free radial forearm flap (n=1) |
| **Flap Trimmed after ICGFA** | 2 | 2 |
| **Post-operative complications** |  |  |
| **Partial flap necrosis** | 0 | 0 |
| **Total flap necrosis** | 0 | 0 |
| **Oral continence** | 0 | 1 |
| **Seroma** | 1 | 0 |
| **Dehiscence** | 1 | 0 |
| **Donor site haematoma** | 1 | 0 |


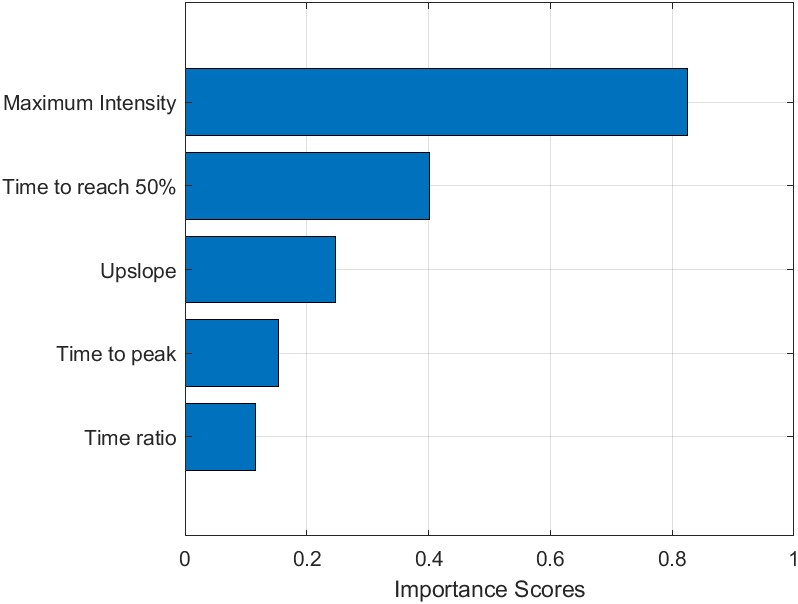


Figure S1. Feature importance scores sorted using ReliefF algorithm.
